# Transfer of oral bacteria to the fetus during late gestation

**DOI:** 10.1038/s41598-020-80653-y

**Published:** 2021-01-12

**Authors:** Kevin Yu, Michelle Rodriguez, Zubin Paul, Elizabeth Gordon, Tongjun Gu, Kelly Rice, Eric W. Triplett, Maureen Keller-Wood, Charles E. Wood

**Affiliations:** 1 Department of Physiology and Functional Genomics, University of Florida College of Medicine, 1345 Center Drive, Room M552, Gainesville, FL 32610 USA; 2 Department of Microbiology and Cell Science, University of Florida Institute of Food and Agricultural Sciences, Gainesville, USA; 3 Department of Pharmacodynamics, University of Florida College of Pharmacy, Gainesville, USA; 4 Interdisciplinary Center for Biotechnology Research, University of Florida, Gainesville, USA

**Keywords:** Reproductive biology, Paediatric research

## Abstract

The fetus develops in a privileged environment, as the placenta serves as both a gateway for nutrients and a barrier for pathogen transfer to the fetus. Regardless, recent evidence suggests the presence of bacterial DNA in both placenta and fetus, and we have reported that DNA and protein from small numbers of bacteria gain access to the fetus from the maternal bloodstream. Other routes of environmental bacterial transfer from the mother to fetus remain unknown, as well as the physiological relevance of their presence. In these experiments, we examine multiple routes by which bacterial cellular components can enter the fetus and the fetal response to influx of bacterial DNA and protein. We inoculated maternal sheep with genetically-labeled *S. aureus* (*Staphylococcus aureus*) using three routes: intravenously, orally, and intra-vaginally. The inoculum did not produce sepsis or fever in the ewes, therefore mimicking incidental exposure to bacteria during pregnancy. 3–5 days post inoculation, we assessed the presence of bacterial components in the fetal tissues and analyzed fetal brain tissue to identify any alterations in gene expression. Our results demonstrate that components of bacteria that were introduced into the maternal mouth were detected in the fetal brain and that they stimulated changes in gene expression. We conclude that an oral route of transmission is relevant for transfer of bacterial cellular components to the fetus.

## Introduction

The fetus develops and grows in a privileged environment. Whereas oxygen, carbon dioxide, and many nutrients traverse the placenta, the placenta represents a relative barrier to transfer of microbes and cells from mother to fetus. In pregnancies not complicated by maternal infection, the fetus is sterile, as evidenced by an inability to culture microorganisms from fetal tissues or fluids^1,2^. Exceptions to this rule of isolation and sterility are few but have been reported in the literature. For example, preterm and full-term infants sometimes have culturable bacteria in meconium, the first stool of the neonate^3–5^; nevertheless, it is not known if some of these pregnancies were complicated by subclinical maternal infection. Another example of exceptions to the exceptionalism of the fetus is the discovery of both maternal and fetal microchimerisms, detected as maternal immune cells in fetal circulation^6,7^ and detection of fetal immune cells in maternal circulation^8^.

We have reported the surprising discovery that bacteria can enter the fetus after transient maternal hypoxia^9^. In several studies, we found that isobaric maternal ventilatory hypoxia stimulated an inflammation-like response in the fetal brain^10,11^ and kidneys^12^ that correspond to the transcriptomics and cellular response that would be expected after bacterial infection. Indeed, culture and nonculture-based techniques confirmed the appearance of bacteria in the fetal brain, and confirmed that the bacteria matched those found in the placenta^9^. Those experiments were performed using the chronically instrumented fetal sheep preparation. While the appearance of bacteria in the brain of the fetus was not explained by the catheterization, the background exposure of the pregnancy (and therefore the presence of live bacteria in the placenta) may have been increased by the chronic catheterization. In summary, these experiments were surprising to us (and others) because of the knowledge that the placenta normally provides an effective barrier to bacterial transfer and therefore maintains sterility of the fetus.

To test whether bacteria—perhaps more accurately stated as bacterial cellular components—can traverse from mother to fetus, we administered small numbers (300 colony forming units, CFU) of a laboratory strain of *S. aureus*, engineered in the laboratory to express fluorescent proteins, intravenously to uncatheterized pregnant sheep. The goal was to test whether there is a hematogenous route by which the fetus can be exposed to bacteria or bacterial components. We reported that, indeed, we could identify fluorescent protein in the fetus using two techniques (immunohistochemistry and ELISA), and that we could detect the presence of the DNA plasmid sequence in the fetus^13^. Having used multiple PCR primers to detect plasmid, our results were consistent with a variable amount of degradation of the plasmid DNA recovered from the fetus^14^. Notably, we could not culture any bacteria—labelled or unlabeled—from the fetal tissues, indicating that the fetus remained sterile^15^.

The present study was performed to address an important gap in our understanding of whether bacteria can ultimately gain access to the fetus from the environment. A relatively common site of infection that can gain access to the fetus is ascending infection in the reproductive tract from vagina^16^. Indeed, this is thought of as being an important cause of premature birth^17–19^ and may be the ultimate source of bacteria found in the meconium of babies born prematurely^19^. Another common source of infection is the maternal mouth^20^. Gingivitis is associated with pregnancy in pregnant women^21–23^; tooth brushing can release bacteria into the bloodstream of pregnant women^24,25^. *P. gingivalis*, a commensal oral bacterial in human, has been found in human placenta^26^, and may be associated with increased risk of prematurity^22^. Tooth brushing, scaling, or flossing in subjects with or without gingivitis have been reported to produce a transient bacteremia with a diverse flora that is at least partially representative of the oral flora^27–29^. To address the question of entry of bacteria into the maternal blood and fetal tissues from the environment, we used the uncatheterized pregnant sheep model to test whether protein or DNA from bacteria placed either into the maternal mouth or into the maternal vagina could be found in the fetus. Finally, to address the question of whether the appearance of bacterial protein and DNA in the fetus is consequential or phenomenological, we tested fetal brain tissue for evidence of a transcriptomics response to maternal inoculation.

## Materials and methods

### Ethical approval

All experiments and procedures were performed according to protocols (201,508,915 and 201,401,710) that were approved by the University of Florida Institutional Animal Care and Use Committee and were performed in accordance with the Guiding Principles for the Use of Animals in Research and Teaching of the American Physiological Society^30^.

### Animals

The animals used in all studies were late-gestation pregnant ewes of mixed (primarily Dorset) breeds. The results reported herein are from two studies: (1) identification of transfer of bacteria from maternal mouth, vagina, or blood to the fetus; and (2) identification of a cellular transcriptomics response in the fetal brain to the transfer of bacteria from mother to fetus.

In *study 1*, we studied two groups of n = 13 and n = 14 fetuses. One group was inoculated with 30,000 CFU of genetically engineered *S. aureus* bacteria expressing Green-, Red-, and Far Red Florescent Protein (GFP, RFP, and FP650; 10,000 CFU each) as described previously^13^. The first group consisted of 6 pregnant sheep carrying twins (n = 5) or triplets (n = 1). The second group (“naïve”) was not inoculated and consisted of 7 pregnant sheep carrying twin fetuses.

In *study 2*, we used tissues from both groups in *study 1* and added a third group which consisted of 7 fetuses whose mothers were inoculated with 300 CFU of a mixture of the same GFP, RFP, and FP650 expressing bacteria, as previously described^13^.

### Experiments

In *study 1*, inoculations were performed on 128–132 days gestation (term in sheep is normally approximately 147 days). Each ewe received a total of 30,000 CFU labeled bacteria administered in three sites. Bacteria administered intravenously (GFP-labelled, 10,000 CFU) were suspended in saline prior to transdermal injection into the exterior jugular vein. Bacteria placed into the maternal mouth (RFP-labelled, 10,000 CFU) were mixed with corn meal, which the sheep find palatable. Bacteria placed into the maternal vagina (FP650-labelled, 10,000 CFU) were suspended in a pharmaceutical compounding base (Versabase gel, PCCA, Houston, TX), which was not lethal to the bacteria, but which provided viscosity that was useful for maintaining retention in the vagina. *Study 2* included tissues from the experiments in *study 1*, plus tissues from previous experiments in which smaller numbers of bacteria were injected into maternal blood^13^. These experiments were performed using pregnant sheep of similar gestational age as described previously^13^.

In both studies, animals were given free access to food and water. Food intake was recorded daily. Blood samples were collected daily (from external jugular vein using a transdermal needle insertion) for blood smears to confirm success of inoculation of bacteria in blood. Ewes were humanely euthanized 3–5 days post inoculation to give bacteria sufficient time to transfer, but reducing time allowed for potential bacterial clearance by the immune system. Various tissues were collected during necropsy. Tissues were either flash frozen with liquid nitrogen for DNA and protein analysis, chilled on wet ice and immediately sent for culturing analysis, or fixed in 4% paraformaldehyde overnight (followed by dehydration and paraffin embedding) for histological analysis. These methods of specimen collection and preservation have been previously described^10,13,31^. The time course of inoculation, blood sampling, and euthanasia/tissue collection in study 2 was similar to that of study 1^13^. Bacteria from tissues were cultured on plates as previously described^13^.

### DNA

DNA was extracted from tissue samples using phenol:chloroform as previously described^13^. Plasmids encoding fluorescent proteins were amplified by endpoint PCR using primers designed by NCBI primer BLAST (Table 1). PCR products were separated according to mass using agarose gel chromatography and visualized using ethidium bromide fluorescence under UV light. Positive samples were counted if product could be visualized and if the length matched that of the positive control. Chi-squared analysis was used to assess significant difference between inoculated samples with naïve samples.

**Table 1:** Primer sequences used for qPCR validation of differential expression of selected genes.

Gene name	FW primer	FW Tm	RV primer	RV Tm	Product length
CAH5	GGCCCCTTGGAAAACCACTA	59.89	AGCGTTCCAGTGAACCAAGT	59.82	132
VAMP1	CTTTCATTTGCACTCGGTCCC	59.8	GATGCCCGCTGTTGTTTACC	59.83	88
COL4A1	GTCCATGATGAGCTGCCTGT	60.11	TTTCTGGTGGGTCTCACGTC	59.61	110
TENA	AAAGACTTGGCCCCACCATC	60.25	GGATGTTGATGCGGTGAGTG	59.27	97
PDYN	CTACGGGGGATTCTTACGGC	59.97	GAGACCGAGTGACCACCTTG	60.04	104

### Protein

Tissue concentrations of Green Fluorescent Protein was assayed using a commercially available ELISA kit (Abcam #ab171581, Cambridge, UK). Frozen samples of fetal cerebral cortex (150–250 mg) were placed into conical centrifuge tubes containing 600 mL cell extraction buffer and homogenized using mortar and pestle. After 20 min of incubation on ice, the samples were centrifuged at 18,000×*g* for 20 min, after which time protein was precipitated from the supernatant using acetone. After acetone precipitation, the pellet was centrifuged at 18,000×*g* for 20 min and the supernatant was removed. The remaining acetone was evaporated from the pellet for 20 min, then resuspended in assay buffer. The protein content of the pellet was measured using the Bradford method^32^. For immunohistochemistry, tissues were fixed in 4% paraformaldehyde overnight, then stored in 70% reagent alcohol until they were embedded. Tissues were cut and embedded in paraffin blocks as previously described^13^. Histological slides were cut at 5–7 µM thickness using a Microm HM 325 (Thermo Fisher Scientific, Kalamazoo, MI) and mounted on glass slides. Nonspecific binding was blocked using BLOXALL (Vector Laboratories, Burlingame CA) for 10 min. GFP was probed using rabbit polyclonal anti-GFP antibody (Millipore AB3080, MilliporeSigma, Burlington, MA) at a 1:500 dilution (2 µg/ml) as previously described^13^. RFP was probed using rabbit polyclonal anti-RFP antibody (ab62341, ABCAM, Cambridge, UK). GFP and RFP immunoreactivities were visualized as fluorescence of VRDye 490 Goat anti-Rabbit IgG (Part#: 926-49120, LI-CORP, Lincoln, NE), used as a second antibody. Double staining of GFP or RFP with *S.aureus* was performed by probing *S. aureus* using mouse monoclonal anti-*S. aureus* antibody [704] (ab37644, ABCAM, Cambridge, UK), and using immunofluorescence by binding VRDye 549 Goat anti-Mouse IgG (Part#: 926-54110, LI-CORP, Lincoln, NE).

### RNAseq

RNA was extracted from 20 fetal cerebral cortices for RNAseq analysis. RNAseq was run using Illumina HiSeq X10 (Illumina, San Diego) with PE2 × 150 bp reads. Initial reads were assessed using FastQC to check for presence of Illumina adapters^33^. Reads were cleaned using BBMap kit (Joint Genome Institute, Berkeley) to remove adapter sequences, rRNA reads, reads with Q score below 20, and reads below the length of 40 bp. De novo transcript assembly and annotation was performed as described by Conesa et al.^33^. Filtered sequences were mapped to the sheep genome Oar_v4.0 by STAR. Mappable reads were assembled using Trinity^34^. Trinotate was used for annotation^35^. Bowtie2 was used to map filtered reads back to assembled transcripts^36^.

### Differential gene (DE) expression analysis

The low quality reads were filtered using Trimmomatic before gene expression quantification^37^. Gene expression was obtained using RSEM with default settings^38^. The expected read counts from RSEM were extract for differential analysis using edgeR^39^. The significantly differential expressed genes were called at thresholds of FDR 0.05. Prior to the DE analysis, PCA was performed to identify outlier samples. No obvious outlier samples were found. The heatmap of differentially expressed genes was generated using R package gplots^40^.

### Gene ontology, pathway, and network analysis

The list of differentially expressed genes (DEG) was used for further gene ontology analysis. We performed gene ontology analyses using two strategies. We first used the list of differentially expressed genes (n = 60 or 53 depending on specific pairwise comparison) using parameters listed above (false discovery rate, FDR < 0.05). Gene names were deduced from their Trinity ID sequences using BLAST and annotation was confirmed using Genecards.org^41^. Gene ontology was analyzed using two methods: (1) Biological Network Gene Ontology Tool (BINGO, Toronto) within the Cytoscape environment^42,43^; and (2) gene ontology analysis within the WebGestalt environment^44^. Pathway analysis was performed using Pathway Commons, within the WebGestalt environment^44^. Network analysis was done using Genemania plugin within the Cytoscape environment^45^.

### Validation of differential analysis

Five genes were selected for validation of differential gene expression analysis. Genes were chosen by the following standards: FPKM value > 10, DE p-value < 0.05, and no replicates throughout the gene list (including different isoforms). The genes CAH5A (Carbonic Anhydrase 5A), VAMP1 (Vesicle Associated Protein 1), COL4A1 (Collagen Type 4 Alpha 1), TNC (Tenascin C), and PDYN (Prodynorphin) were chosen using the restraints as listed above. Primers were designed using primerBLAST by NCBI and synthesized by SigmaAldrich (SIGMA, St. Louis)^46^ and are reported in Table 1. Relative gene expression was quantified using qPCR with FAST SYBR green reporter (Cat# 4385614) (ThermoFisher, Waltham). β actin (ACTB) mRNA abundance was used as a housekeeping gene for the purpose of normalization. Results are reported as values of dCt (difference in cycle thresholds of target gene and ACTB). Student’s t-test or Mann–Whitney U test was used to determine significance with threshold of *p* < 0.05^47,48^.

## Results

### Physiological response to inoculation

Animals inoculated with 10,000 CFU *S. aureus* expressing GFP, RFP, or FP650 each at three different sites, respectively intravenous, oral, and vaginal, exhibited no signs of sepsis, fever, or anorexia. Body temperatures were maintained within normal physiological limits (102.0 ± 0.5° F, mean ± SD, n = 38)^49^. Feed and hay consumption were recorded daily and showed no significant change post inoculation (data not shown). We were unable to detect GFP, RFP, or FP650 expressing *S. aureus* in blood smears drawn daily from jugular vein (starting 1 day after inoculation).

### DNA

PCR of plasmid DNA detected all three plasmids in fetal liver (p < 0.05, Table 2). We could also detect GFP and RFP plasmids in fetal cerebral cortex, cotyledon, and spleen (*p* < 0.05, Table 2). FP650 plasmid was detectable in fetal liver (*p* < 0.05, Table 2), but not reliably detected in cotyledon (*p* = 0.07, Table 2), fetal spleen, or cerebral cortex (*p* > 0.05, Table 2).

**Table 2:** PCR of fluorescent plasmids counted by presence of bands after gel electrophoresis.

Liver	Inoculated positive	Inoculated negative	Naïve positive	Naïve negative	Significance Chi-square
GFP plasmid	15	1	0	14	*p* = 1.96e−06
RFP plasmid	10	6	0	14	*p* = 0.001
FP650 plasmid	6	10	0	14	*p* = 0.04
**Cortex**					
GFP plasmid	14	2	0	7	*p* = 0.0005
RFP plasmid	14	2	0	14	*p* = 1e−05
FP650 plasmid	0	16	0	7	*p* = NA
**Spleen**					
GFP plasmid	14	0	0	8	*p* = 0.0002
RFP plasmid	8	6	0	14	*p* = 0.003
FP650 plasmid	1	13	0	14	*p* = 1
**Cotyledon**					
GFP plasmid	15	1	0	7	*p* = 0.0001
RFP plasmid	16	0	0	11	*p* = 2e−06
FP650 plasmid	5	11	0	14	*p* = 0.07

Chi-squared analysis to test for significant difference compared to naïve controls.

### Protein

**Figure 1:**
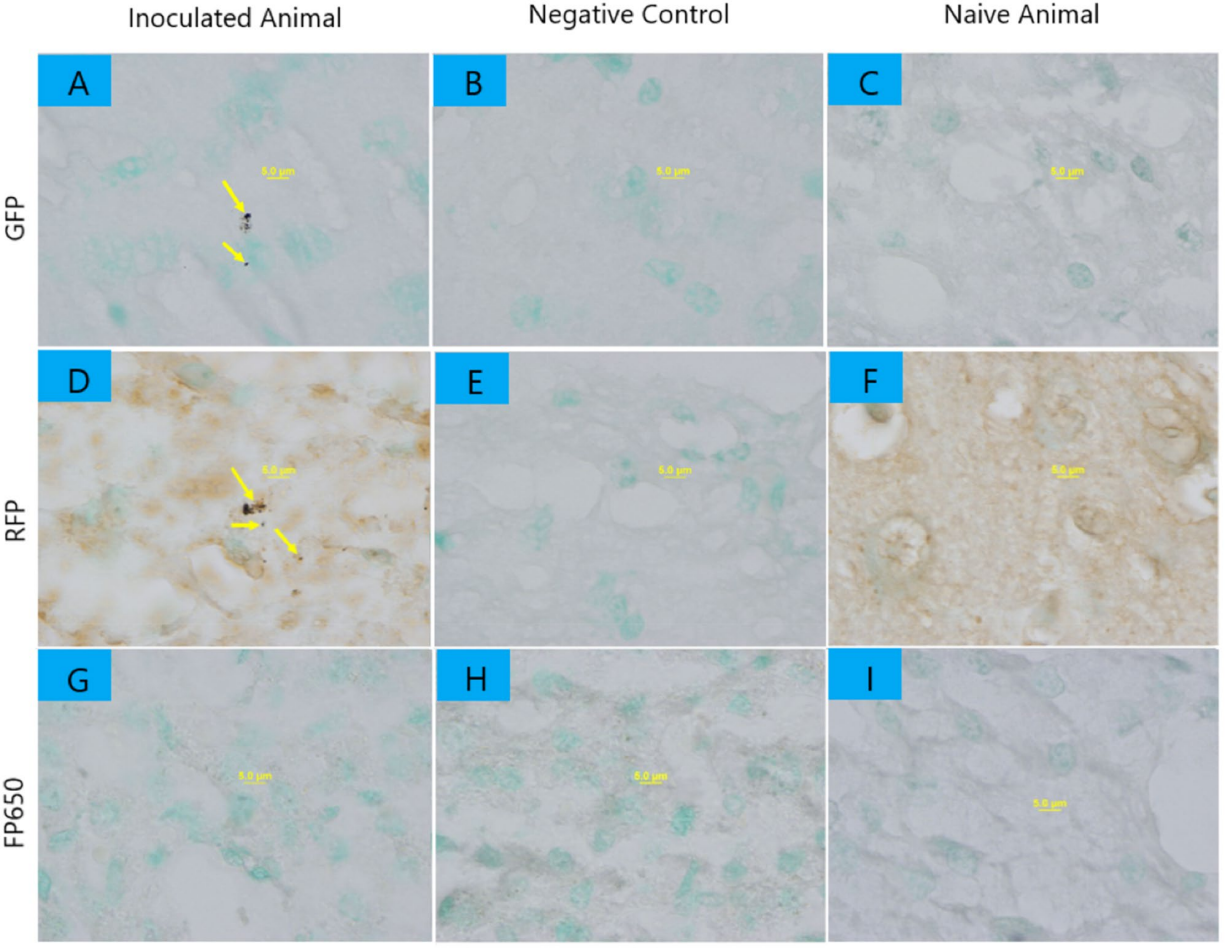
Bacteria fluorescent protein IHC staining with DAB reporter (dark brown) of fetal cerebral cortex. ×100 magnification. (**A**) Inoculated animal, GFP immunohistochemical labeling of bacteria (yellow arrows); (**B**) negative control, immunohistochemical labeling without primary (anti-GFP) antibody. (**C**) Naïve animal, GFP immunohistochemical labeling as in (**A**); (**D**) inoculated animal, RFP immunohistochemical labeling of bacteria (yellow arrows); (**E**) negative control, immunohistochemical labeling without primary (anti-RFP) antibody. (**F**) Naïve animal, RFP immunohistochemical labeling of bacteria. (**G**) Inoculated animal, FP650 immunohistochemical labeling of bacteria; (**H**) Negative control, immunohistochemical labeling without primary (anti-FP650) antibody. (**I**) Naïve animal, RFP immunohistochemical labeling of bacteria.

**Figure 2:**
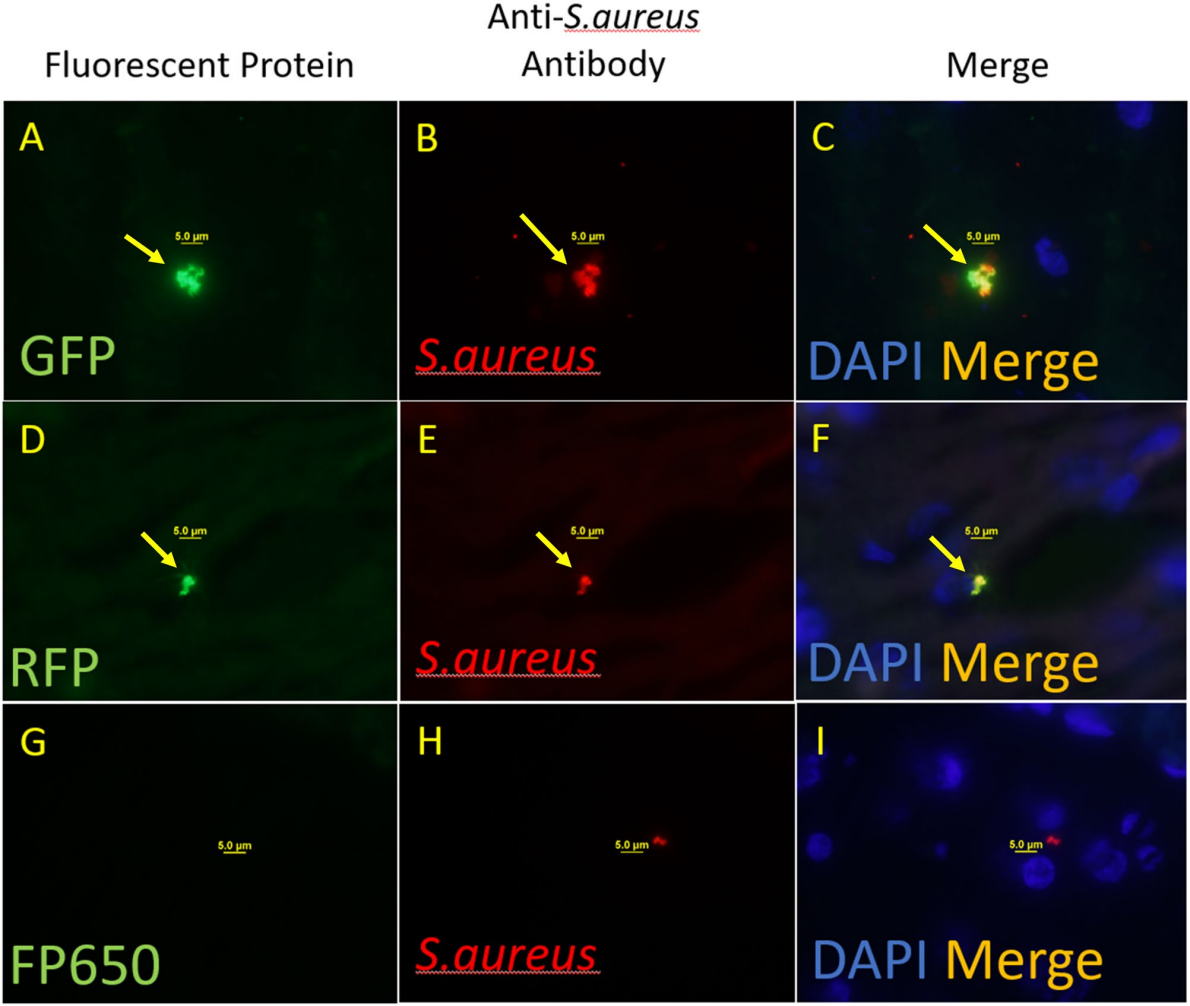
Fetal cerebral cortex double immunofluorescence stain with anti-fluorescent protein antibody (green fluorescence), anti-*S.aureus* bacteria (red fluorescence), and DAPI in blue. ×100 magnification: (**A**) GFP (Green), (**B**) *S. aureus* (Red), (**C**) Merge images of GFP staining. (**D**) RFP (Green), (**E**) *S. aureus* (Red), (**F**) Merge images of RFP staining. (**G**) FP650 (Green), (**H**) *S. aureus* (Red), (**I**) Merge images of FP650 staining.

We detected fluorescent proteins at the protein level using ELISA, immunohistochemistry, and immunofluorescence. Using immunohistochemistry with DAB as reporter, we observed small groups of GFP- and RFP-immunoreactive coccus-shaped bacteria of the same approximate size as *S. aureus* in fetal cerebral cortex (Fig. 1). Cerebral cortex from naïve controls exhibited no specific staining. We were not able to find FP650-immunoreactive bacteria in fetal cerebral cortex from inoculated animals (Fig. 1). Double immunofluorescence staining of fetal cerebral cortex with anti-GFP or RFP antibody (green) and anti-*S. aureus* antibody (red) showed overlap of GFP and RFP and *S. aureus* (Fig. 2). Double immunofluorescence staining of FP650 and *S. aureus* showed only positive stain of *S. aureus* with marginally detectable staining of FP650 (Fig. 2).

**Figure 3:**
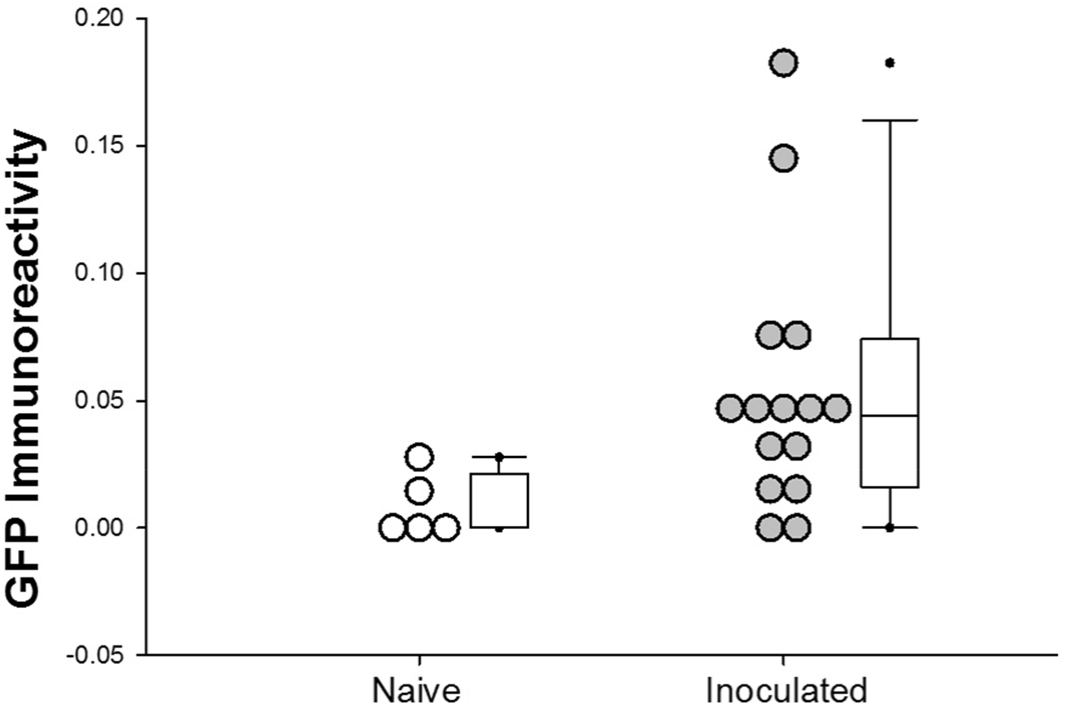
GFP ELISA of fetal cerebral cortex. Results of ELISA for GFP immunoreactivity of fetal cerebral cortex in animals whose mothers received an intravenous inoculation of 10,000 CFU of GFP-labeled *S. aureus* intravenously. Box and whisker plot with median (horizontal line) denoted, boundaries of box indicate 25th and 75th percentiles. Individual data are shown as open (naïve: uninoculated) or filled (inoculated) symbols. Whiskers (error bars) indicate 90th and 10th percentiles. *p < 0.05.

We successfully detected immunoreactive GFP in fetal cerebral cortex using ELISA. The abundance of immunoreactive GFP was significantly greater in fetal cerebral cortex from inoculated sheep compared to sheep that were not inoculated (Fig. 3). The presence of ELISA-measurable GFP protein in fetal cerebral cortex confirms our previous report of similar findings with a lower dose of inoculum introduced into the maternal blood^13^.

### Culture

We were unable to culture live plasmid-containing bacteria from fetal tissues or blood. Culture plates showed no bacterial growth after 48 h of incubation.

### RNAseq

Analysis of differentially expressed genes in the fetal cerebral cortex revealed that the small, subclinical, bacterial load administered to pregnant sheep results in changes in gene expression in the fetal cerebral cortex. When comparing naïve fetal cerebral cortices to those in animals that received 300 CFU of fluorescent protein-expressing *S. aureus* into the maternal blood, we identified 59 differentially expressed genes (DEG: 36 upregulated and 23 downregulated). When comparing non-inoculated animals to the animals that received the larger dose of 30,000 CFU (10,000 each to maternal blood, mouth, and vagina) we identified 75 DEG (38 upregulated and 37 downregulated).

We validated the identity of genes originally identified from TRINITY sequences using qPCR with primer sequences designed to anneal within their coding regions. All five genes selected for analysis were significantly differentially expressed with *q* < 0.05 as analyzed using qPCR (Table 3).

**Table 3:** Quantitative PCR analysis of a sample of differentially-expressed genes.

Gene	naïve (dCt ± SEM)	300 CFU (dCt ± SEM)	30,000 CFU (dCt ± SEM)
CAH5H	16.4 ± 0.4 (n = 5)	6.2 ± 2.5* (n = 5)	10.3 ± 2.1* (n = 7)
VAMP1	4.8 ± 0.1 (n = 6)	5.6 ± 0.2* (n = 7)	5.4 ± 0.2* (n = 7)
COL4A1	4.4 ± 0.2 (n = 6)	3.9 ± 0.1 (n = 7)	3.8 ± 0.1* (n = 7)
TNC	7.0 ± 0.1 (n = 6)	6.2 ± 0.2* (n = 7)	6.1 ± 0.2* (n = 7)
PDYN	5.9 ± 0.9 (n = 6)	7.4 ± 0.2^#^ (n = 7)	6.5 ± 0.7 (n = 7)

*p* < 0.05 versus Naïve.

**Figure 4:**
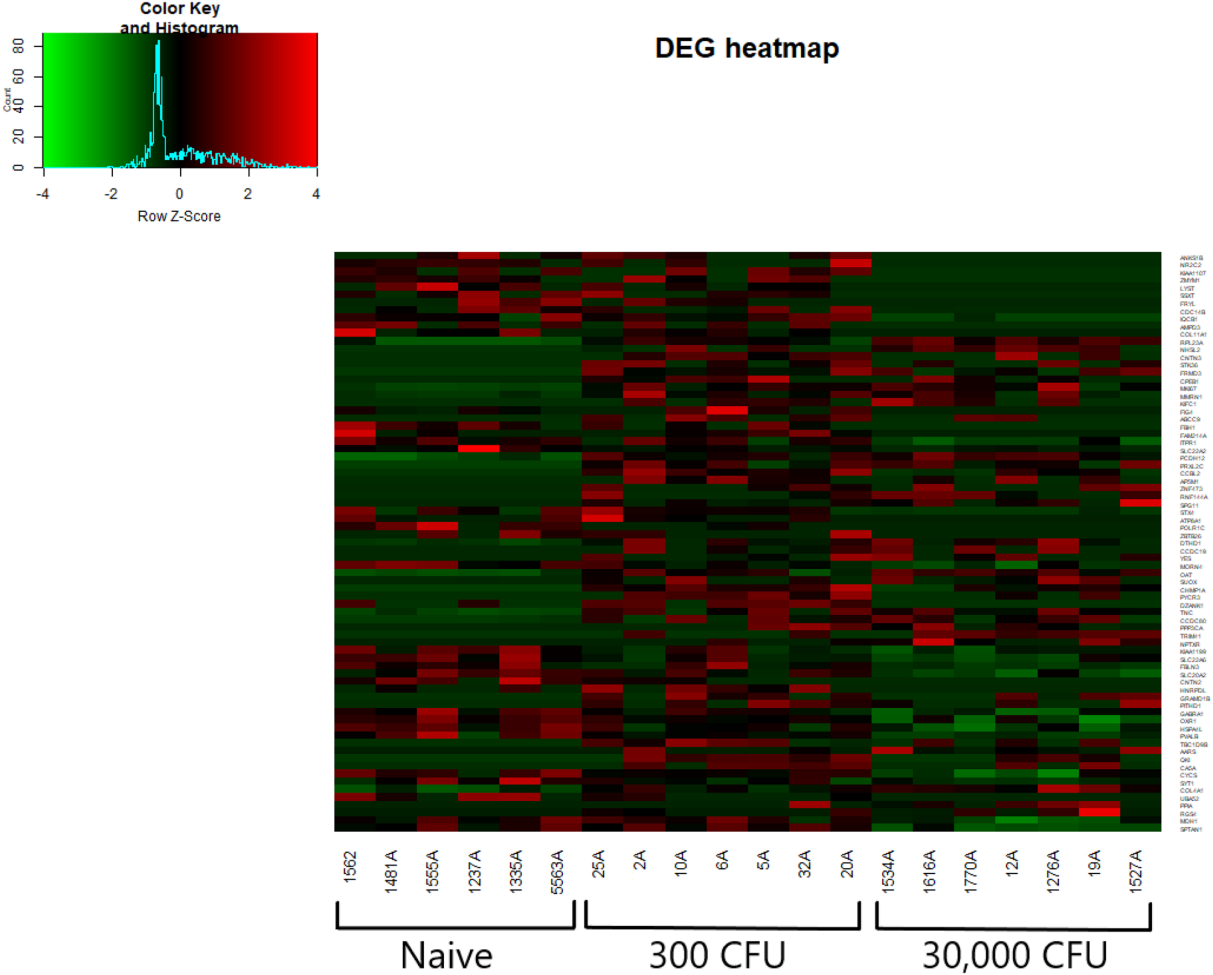
DEG heatmap with animals and animal groups labeled. Clear separation between groups should be noted. Heatmap generated using fragments per kilobase of transcript per million mapped reads (FPKM) value. Higher values indicated in red represents higher expression. Key indicates frequency of FPKM values based on Z-score or number of standard deviations above or below the mean.

**Figure 5:**
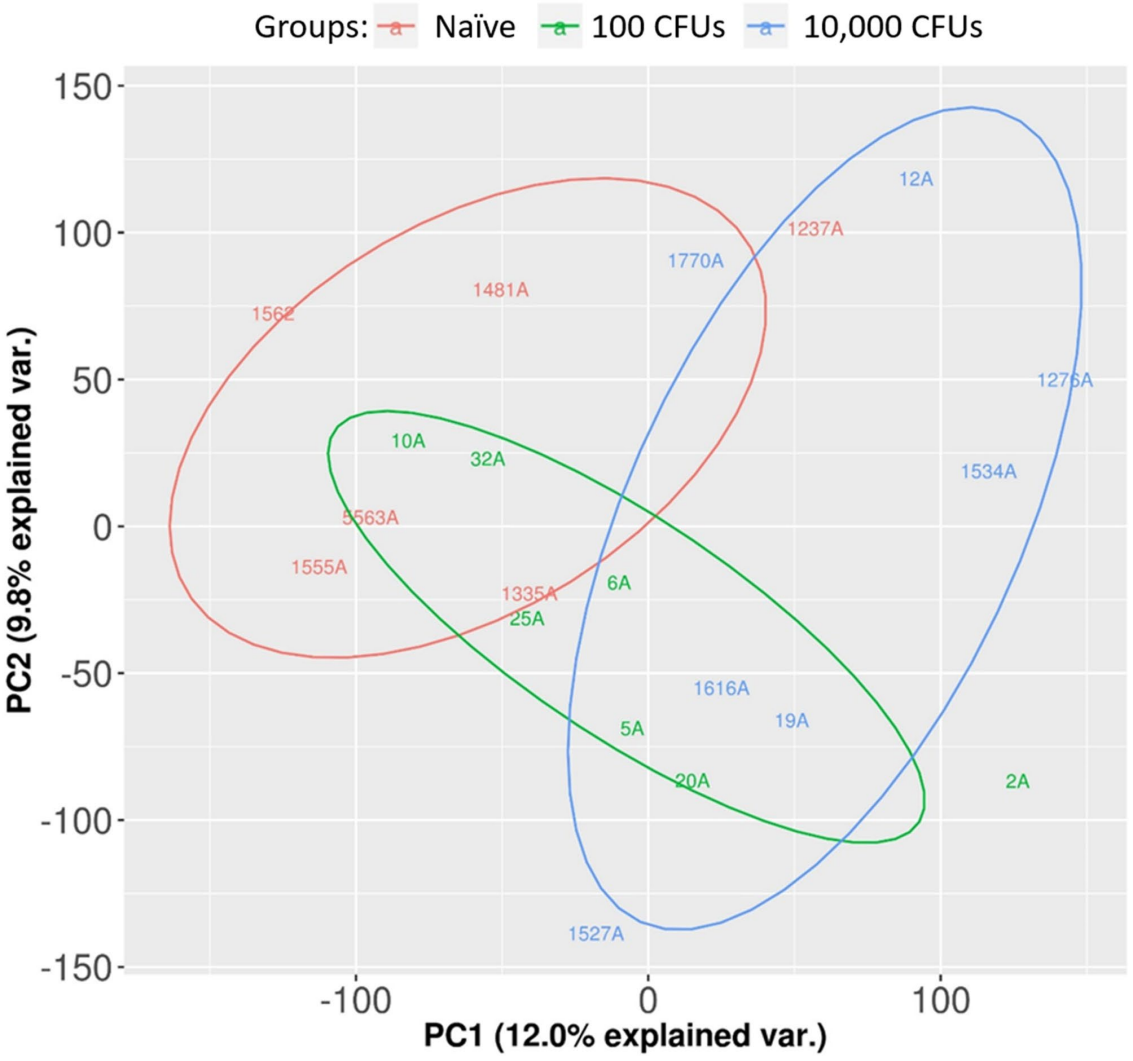
Principal Component Analysis of differentially expressed genes in the three experimental groups. Naïve control animals in red, 300 CFU inoculation group in green, and 30,000 CFU inoculation group in blue. Identifiers represent animal numbers.

**Figure 6:**
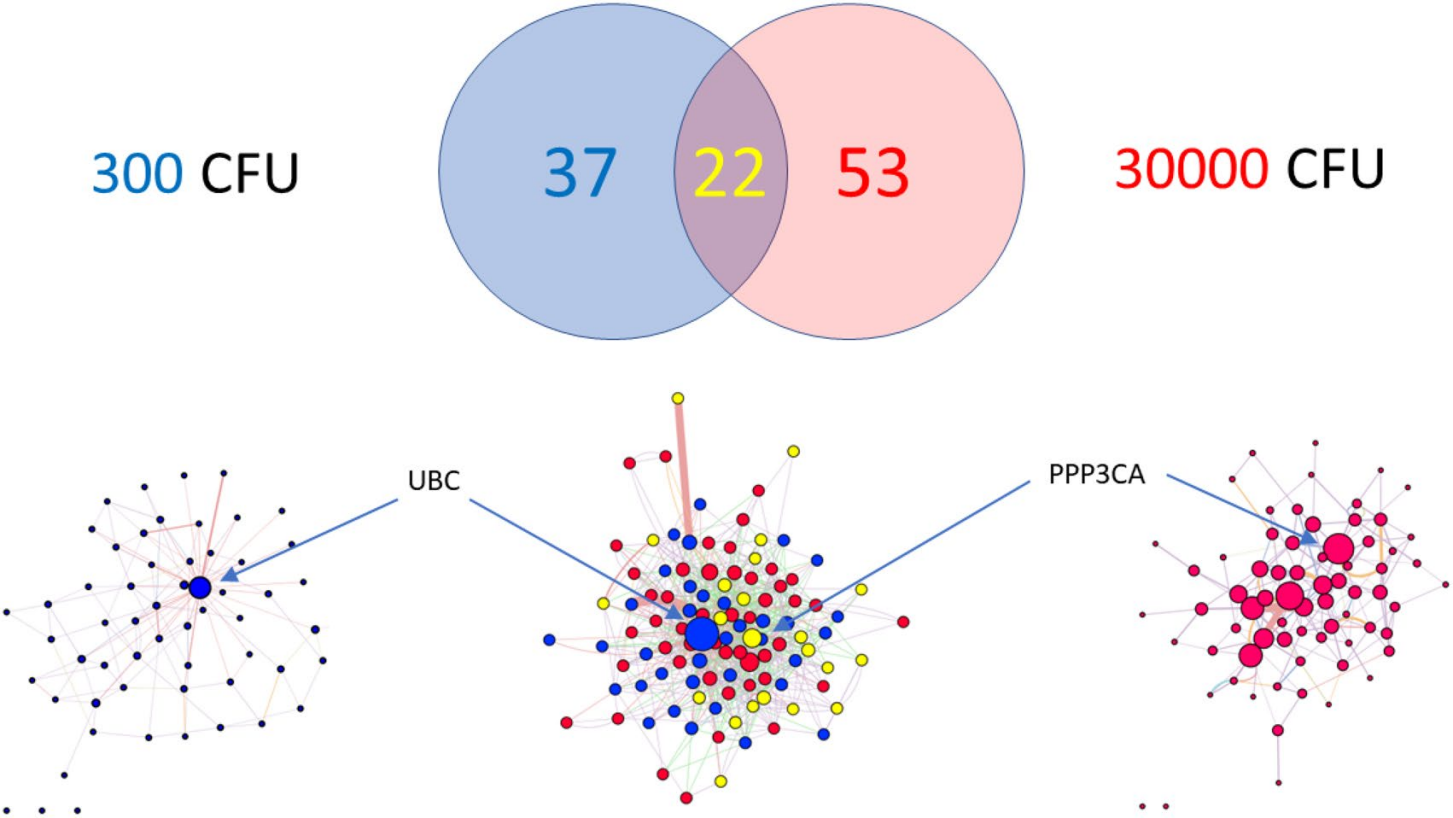
Top: Venn diagram of DEG in 300 and 30,000 CFU experiments. Bottom: Networks of DEG for 300 CFU (left), 30,000 CFU (right) experiments. Network of both sets of DEG is shown in bottom, center. Network nodes colored blue, red, and yellow denote genes in 300, 30,000, and both 300 and 30,000 DEG gene sets, respectively. The sizes of the nodes are scaled to the calculated parameter “stress”; therefore, larger nodes are more connected to other nodes in the network.

Differential expression is illustrated by the heatmap of DEG shown in Fig. 4 and by the relative separation of groups in the Principal Component Analysis (Fig. 5). As shown in Fig. 6, 22 genes were differentially expressed as the result of both treatments, 37 genes were specific to the lower dose and 53 genes were specific to the higher dose.

Gene ontology analyses using WebGestalt, with Benjamani-Hochburg correction did not yield statistically significant association of either set of DEG with annotated Biological Processes. Pathway analysis of DEG for both sets of DEG by PathwayCommons, however, revealed statistically significant association with known pathways (Table 4). DEG in 300 CFU experiments revealed significant association with pathways that included: *Internalization of ERBb1*, *Beta1 integrin cell surface interactions*, and *Arf6 signaling events*. DEG in the 30,000 CFU experiments revealed significant association with pathways that included *Organic cation/anion/zwitterion transport*, *Transmembrane transport of small molecules*, and *Beta3 integrin cell surface interactions*.

**Table 4:** Pathway commons analysis.

Pathway name	Genes	Adjusted *p* value
**(A) 300 CFU inoculation experiments**		
Internalization of ErbB1	PPP3CA, CLIP1, ASAP2, RCC1, CLTC, NDRG2, YES1, TBL1X	*p* = 0.0003
arginine degradation VI (arginase 2 pathway)	PYCR3, OAT	*p* = 0.0003
Signaling events mediated by VEGFR1 and VEGFR2	PPP3CA, CLIP1, ASAP2, RCC1, CLTC, NDRG2, YES1, TBL1X	*p* = 0.0003
Beta1 integrin cell surface interactions	PPP3CA, CLIP1, ASAP2, RCC1, CLTC, NDRG2, YES1, TBL1X, TNC	*p* = 0.0003
Urokinase-type plasminogen activator (uPA) and uPAR-mediated signaling	PPP3CA, CLIP1, ASAP2, RCC1, CLTC, NDRG2, YES1, TBL1X	*p* = 0.0003
PDGFR-beta signaling pathway	PPP3CA, CLIP1, ASAP2, RCC1, CLTC, NDRG2, YES1, TBL1X	*p* = 0.0003
Arf6 trafficking events	PPP3CA, CLIP1, ASAP2, RCC1, CLTC, NDRG2, YES1, TBL1X	*p* = 0.0003
Arf6 signaling events	PPP3CA, CLIP1, ASAP2, RCC1, CLTC, NDRG2, YES1, TBL1X	*p* = 0.0003
Signaling events mediated by focal adhesion kinase	PPP3CA, CLIP1, ASAP2, RCC1, CLTC, NDRG2, YES1, TBL1X	*p* = 0.0003
LKB1 signaling events	PPP3CA, CLIP1, ASAP2, RCC1, CLTC, NDRG2, YES1, TBL1X	*p* = 0.0003
**(B) 30,000 CFU inoculation**		
Organic cation/anion/zwitterion transport	SLC22A2, SLC22A6	*p* = 0.0047
Norepinephrine neurotransmitter release cycle	SLC22A2, SYT1	*p* = 0.0047
Transmembrane transport of small molecules	SLC22A2, ABCC9, ATP8A1, SLC20A2, SLC22A6	*p* = 0.0095
Neurotransmitter release cycle	SLC22A2, SYT1	*p* = 0.0166
Response to elevated platelet cytosolic Ca2+	STX4, PPIA	*p* = 0.025
Neuronal system	SLC22A2, ABCC9, SYT1	*p* = 0.025
Gene expression	UBA52, ZNF473, AARS1, RPL23A	*p* = 0.025
Metabolism of amino acids and derivatives	KYAT3, UBA52, OAT	*p* = 0.025
Beta3 integrin cell surface interactions	COL4A1, TNC	*p* = 0.025
Caspase cascade in apoptosis	CYCS, SPTAN1	*p* = 0.025

Tables report the top 10 pathways over-represented in 300 (A) and 30,000 (B) CFU inoculation experiments.

Each set of DEG was associable into individual networks using guilt-by-association analysis^50–52^, as shown in Fig. 6. Thirty four of the 37 DEG’s after 100 CFU inoculation were all associable into a single network (74% physical interaction; 23% co-expression; 2% predicted; and 1% genetic interaction. Hierarchicalization of the network revealed that the entire network of DEG could be related to a single gene: ubiquitin C (UBC). This is confirmed by analysis of this network using the CentiScape plugin module of Cytoscape, showing that the gene with the highest value of “stress” (a parameter of connectedness) is UBC. The top 10 genes with respect to “stress” were: UBC, YES1, ATP6V1G2, SUSD1, CCK, CNKSR2, AP5M1, GMFB, CCDC115, and TBC1D9B. Fifty one of the 53 DEG’s identified after 30,000 CFU inoculation could be organized into a single network (48% physical interaction; 29% co-expression; 2% co-localization; 18% predicted; and 3% genetic interaction). Hierarchicalization of this network revealed that the highest ranking genes were SYT1 and PPP3CA. The top 10 genes with respect to “stress” were CNTN2, PPP3CA, PPIA, AP5M1, MDH1, GABRA1, SPG11,HSPA4L, ITPR1, CNTN3. Because of the significant overlap between sets of DEG and because of the similarity in associated pathways, the combined sets of DEG could be organized into a single network of associated genes (Fig. 6). Analysis of the centrality of this combined network revealed that the most interconnected genes were Ubiquitin C (UBC) and Protein Phosphatase 3 Catalytic Subunit Alpha (PPP3CA), with the latter node shared between both gene sets.

## Discussion

We demonstrated that, during late gestation in pregnant sheep: (1) DNA and protein from bacteria introduced in maternal mouth, blood, or (to a lesser extent) vagina, appears in placenta and fetal tissues; and (2) the influx of bacteria or bacterial cell components stimulate a cellular response (identified as a change in tissue transcriptomics) in the fetal cerebral cortex.

The present work confirms and extends our recent report that DNA and protein from bacteria introduced into the maternal bloodstream of pregnant sheep can later be found in the fetus^13^. While this result challenges the currently accepted dogma of fetal exceptionalism, it is consistent with existing literature. Recent studies have demonstrated the presence of bacterial DNA in placenta of human beings^53,54^ and of various species, including mouse^20^, rat^55^ and sheep^9,13^. Metagenomics analysis of the bacterial DNA in human placenta suggested that the taxa identified in placenta was similar to that known to be found in maternal mouth^20,53,56–58^. There are several reports of the presence of live bacteria in the meconium of newborn infants, consistent with colonization of the normal fetus prior to birth^13,53,59–61^. Transfer of bacteria to the fetus appears to be increased by maternal and fetal hypoxia, perhaps mediated by hypoxia-induced changes in vascular permeability^9^.

In this and in a previous study, we have shown that transfer of bacterial DNA and protein from mother to fetus can occur with small numbers of bacteria in the mother, too small to mimic maternal infection. Introduction of very low numbers of bacteria into the maternal bloodstream resulted in the appearance of assayable bacterial components in the fetus^13^, although it is not known whether the bacterial cells were alive, dead, or dormant when transferred into the fetus. In previous experiments, we introduced 300 CFU of *S. aureus* bacteria containing GFP, RFP, and FP650-encoding plasmids into the maternal blood intravenously. While we could find protein and DNA from the engineered bacteria, we were unable to demonstrate the presence of live GFP, RFP, or FP650-expressing bacteria in the fetus. That study provided proof of principle that bacteria (either alive, dead, or dormant) can traverse placenta or other barriers into the fetus^13^. The design of the present study represents an extension of that study, with multi-site inoculation of the sheep in blood, mouth, and vagina, and with an increased number of bacteria in the inoculum (30,000 CFU) to allow for appropriate detection sensitivity in the fetus. Although we used a higher dose of inoculum in the present study, the bacteria were cleared without causing transient fever or alteration in maternal behavior that would have been consistent with maternal illness.

Consistent with our previous study, cellular DNA and protein from GFP-expressing bacteria inoculated intravenously were detectable in the fetal organs. In the present study, we found protein and DNA from RFP-expressing bacteria introduced into the maternal mouth appeared in the fetus. The appearance of DNA and protein from RFP-expressing bacteria in the fetus demonstrated that bacteria introduced into the maternal mouth also traversed to the fetus (either alive, dead, or dormant). The route from maternal mouth to placenta and fetus is consistent with observations by other investigators that, in the human, bacterial DNA in placenta matches that reported in the maternal mouth^26,62,63^, oral bacteria are found in human placenta after preterm birth^23^, and that oral inoculation of the pregnant mouse with *P. gingivalis* results in detection of that bacteria in the placenta^21^. Bacteria present in the maternal mouth have been observed in the placenta and have been attributed to be associated with preterm birth^23,26^, suggesting that very large numbers of bacteria can cause clinical infection via the same route and stimulate preterm labor. With regard to transfer of bacteria across placenta, we are mindful of the limitation of the animal model that the sheep placenta has a different structure than the human or nonhuman primate placenta. The sheep placenta is epitheliochorial placenta, while the human is hemochorial. Extrapolation from sheep to human with regard to the transfer of bacteria or their cellular components from mother to fetus is therefore not possible on the basis of the present experiments. There are fewer cellular layers separating fetal and maternal blood in the human placenta, which might suggest the possibility that the sheep placenta affords more protection of the fetus compared to the human placenta. However, other differences in the placental anatomy, for example the existence of a syncitiotrophoblast layer in the human, might suggest that the human placenta affords greater protection. We are also mindful of the current controversy surrounding the metagenomics-based detection of bacterial DNA in normal human placenta^53,64^. Because of the sensitivity of metagenomics-based techniques and the potential susceptibility of shotgun sequencing to contamination artifact, the validity of studies identifying bacterial DNA in human placenta have been questioned^64^. While interesting and relevant to maternal–fetal physiology, the scope of the present study is limited to address the appearance of bacterial cell components in the fetus after inoculation of the mother. The present results do not address whether there is or is not a “placental microbiome” in unmanipulated pregnancies, only whether there is communication from mother to baby—a mode of communication that might bear relevance to immune competence and readiness for birth.

DNA and protein from FP650-expressing bacteria, introduced into the maternal vagina, were less reliably detected. We interpreted these data to indicate either that the route from maternal vagina to placenta and fetus in the sheep is not as facile as originally thought, or that the sensitivity of our PCR technique might have been too low to detect small amounts of potentially fragmented FP650 plasmid DNA. With regard to vaginal inoculation, we are mindful of the limitations of the sheep as a model for transfer of bacteria from the human vagina to the human fetus. The sheep cervix has many unique shapes including spiral, duckbill, flap, rosette, and papilla^65^; whereas the shape of the human cervix is simpler^66,67^. The unique shapes of the sheep cervix have been reported to hinder artificial insemination^68^. The shape of the cervix in this species might possibly hinder bacterial migration from the vagina to fetus allowing only transvascular absorption of bacteria or their components through the vaginal lining^69^. 

To avoid spurious conclusions concerning detection of cellular components of the inoculated bacteria in the fetal tissues, we used several methodologies: (1) plasmid DNA was detected using endpoint PCR followed by gel electrophoresis; (2) expressed fluorescent protein was detected using ELISA (for GFP); and (3) by immunohistochemistry and immunofluorescence. With regard to detection of immunoreactive protein, the use of both DAB and fluorescence as reporters minimized the possibility of false positive detection caused by autofluorescence or endogenous peroxidase activity. DAB detection revealed that bacteria (or digests of bacteria) group into aggregates within the fetal cerebral cortex. We do not know whether the detected immunoreactivity is intracellular or extracellular or, for example, whether they have been engulfed in immune cells. Immunofluorescence visualization confirmed co-localization of fluorescent proteins and *S.aureus* markers. Both GFP and RFP showed clear overlap of anti-GFP/RFP antibody with anti-*S.aureus* antibody binding, providing further confidence in our conclusion that the labeled bacteria (or their remnants) were identifiable in fetal tissues.

With regard to detection of plasmid DNA and of immunoreactive protein, it is possible and perhaps likely that material that we detected is from dead or dormant bacteria^70^. We were unable to culture live fluorescent protein-expressing bacteria from the fetal tissues. We are confident that, because the engineered bacteria are *S. aureus*, and because we have had extensive experience producing and culturing these cells, we would have likely cultured them from fetal tissues if they were alive or not otherwise dormant. Nevertheless, we also recognize that the tissues might have contained very low numbers of widely dispersed live bacteria in tissue.

Both inoculation protocols resulted in statistically significant changes in gene expression in the fetal cerebral cortex illustrating that, in addition to the demonstration of bacterial DNA and protein in the cortical tissue, there is a response of the tissue to the bacterial exposure. Network analysis of the responding genes revealed that the response to both inoculation doses involved highly related gene sets, and that the DEG from both groups are highly related to each other. Hierarchical analysis of the combined networks of DEG indicated that the most highly connected genes—likely the regulatory “hubs” of the networks—are UBC and PPP3CA. The importance of UBC for the gene expression response in the 300 CFU inoculation raises the possibility of increased protein degradation and elimination of protein aggregates^71^. Pathway analysis suggests the possible involvement of ErbB1 internalization and Arf6 signaling. Both of these pathways have been associated with cellular bacterial invasion^72–74^. While the gene expression response to 30,000 CFU overlapped with and therefore appeared related to that of the 300 CFU experiments, the importance of PPP3CA (and of SYT1, which was also a highly connected hub in the network), along with the pathway analysis, suggests that the majority of the response was related to changes in neuron and neurotransmitter function. It is possible that the 30,000 CFU inoculation was of sufficient magnitude to stimulate cellular changes in function within the fetal brain.

We interpret our results as an indication that the fetus is responding to the influx of small numbers of bacteria or bacterial cellular components, not as an indication of infection and inflammation. In our previous study, in which we found evidence of infection-, immune- and inflammation-related gene expression after influx of live bacteria into the fetus, we observed substantial increases in the mRNA abundance of C-X-C Motif Chemokine Ligands 10 and 16 (CXCL10 and CXCL16), Tumor necrosis factor alpha (TNFA), Toll-like receptor 2 (TLR2), and Nuclear Factor kappa-light chain enhancer of activated B cells (NFKB)^10^. Pathway analysis of the transcriptomics response in that study aligned significantly with *Immune System* and *Innate Immune System*. While we did not, in the present study, observe evidence of immune system responses or TLR signaling, we did find some overlap in the pathway analysis related to integrin cell surface and Arf6 signaling pathways. While it is clear that frank infection increases likelihood of preterm birth or intrauterine death^18,75^, much less is known about whether exposure of the fetus to very much lower numbers of bacteria occurs as a feature of normal, physiological, fetal development. We suspect that exposure to bacterial antigens is not uncommon in healthy pregnancy and that the response of the fetal brain tissue is consistent with clearance of dead bacteria from the fetal brain tissue.

In conclusion, our results are consistent with the thesis that the fetus is exposed to cellular components of maternal oral bacteria with eventual transfer to the fetus via the maternal bloodstream to the placenta^26^. While we were unable to recover live bacteria from the fetal brain tissue, we are intrigued by the principle that there is a natural source of communication from environment to fetus. The biological importance of this route of communication is unclear, but may be one method by which the fetus is readied for exposure to the world ex-utero.

## References

[1] Hall IC, O'Toole E (1934). Bacterial flora of first specimens of meconium passed by fifty new-born infants. JAMA Pediatr..

[2] Tissier H (1900). Recherches sur la flore intestinale des nourrissons.

[3] Hansen R (2015). First-pass meconium samples from healthy term vaginally-delivered neonates: An analysis of the microbiota. PLoS ONE.

[4] Stinson LF, Keelan JA, Payne MS (2019). Characterization of the bacterial microbiome in first-pass meconium using propidium monoazide (PMA) to exclude nonviable bacterial DNA. Lett. Appl. Microbiol..

[5] Ardissone AN (2014). Meconium microbiome analysis identifies bacteria correlated with premature birth. PLoS ONE.

[6] Desai RG, Creger WP (1963). Maternofetal passage of leukocytes and platelets in man. Blood.

[7] Hall JM (1995). Detection of maternal cells in human umbilical cord blood using fluorescence in situ hybridization. Blood.

[8] Bianchi DW, Zickwolf GK, Weil GJ, Sylvester S, DeMaria MA (1996). Male fetal progenitor cells persist in maternal blood for as long as 27 years postpartum. Proc. Natl. Acad. Sci. U.S.A..

[9] Zarate MA (2017). Post-hypoxia invasion of the fetal brain by multidrug resistant Staphylococcus. Sci. Rep..

[10] Chang EI (2016). Ketamine decreases inflammatory and immune pathways after transient hypoxia in late gestation fetal cerebral cortex. Physiol. Rep..

[11] Zarate MA, Chang EI, Wood CE (2018). Effects of ketamine on the fetal transcriptomic response to umbilical cord occlusion: Comparison with hypoxic hypoxia in the cerebral cortex. J. Physiol..

[12] Chang EI (2016). Ketamine suppresses hypoxia-induced inflammatory responses in the late-gestation ovine fetal kidney cortex. J. Physiol..

[13] Yu K (2019). Proof of principle: Physiological transfer of small numbers of bacteria from mother to fetus in late-gestation pregnant sheep. PLoS ONE.

[14] Rodriguez MD, Paul Z, Wood CE, Rice KC, Triplett EW (2017). Construction of stable fluorescent reporter plasmids for use in. Front. Microbiol..

[15] Sandle, T. In *Sterility, Sterilisation and Sterility Assurance for Pharmaceuticals* (ed. Sandle, T.) 1–20 (Woodhead Publishing, 2013).

[16] Gundogan F, De Paepe ME (2013). Ascending infection: Acute chorioamnionitis. Surg. Pathol. Clin..

[17] Pararas MV, Skevaki CL, Kafetzis DA (2006). Preterm birth due to maternal infection: Causative pathogens and modes of prevention. Eur. J. Clin. Microbiol. Infect. Dis..

[18] Goldenberg RL, McClure EM, Saleem S, Reddy UM (2010). Infection-related stillbirths. Lancet.

[19] Burdet J (2014). Inflammation, infection and preterm birth. Curr. Pharm. Des..

[20] Fardini Y, Chung P, Dumm R, Joshi N, Han YW (2010). Transmission of diverse oral bacteria to murine placenta: Evidence for the oral microbiome as a potential source of intrauterine infection. Infect. Immun..

[21] Liang S (2018). Periodontal infection with *Porphyromonas gingivalis* induces preterm birth and lower birth weight in rats. Mol. Oral. Microbiol..

[22] Ao M (2015). Dental infection of *Porphyromonas gingivalis* induces preterm birth in mice. PLoS ONE.

[23] Radochova V (2018). Periodontal disease and intra-amniotic complications in women with preterm prelabor rupture of membranes. J. Matern. Fetal Neonatal Med..

[24] Lockhart PB (2008). Bacteremia associated with toothbrushing and dental extraction. Circulation.

[25] Parahitiyawa NB, Jin LJ, Leung WK, Yam WC, Samaranayake LP (2009). Microbiology of odontogenic bacteremia: Beyond endocarditis. Clin. Microbiol. Rev..

[26] Blanc V (2015). Oral bacteria in placental tissues: Increased molecular detection in pregnant periodontitis patients. Oral Dis..

[27] Tomas I, Diz P, Tobias A, Scully C, Donos N (2012). Periodontal health status and bacteraemia from daily oral activities: Systematic review/meta-analysis. J. Clin. Periodontol..

[28] Forner L, Larsen T, Kilian M, Holmstrup P (2006). Incidence of bacteremia after chewing, tooth brushing and scaling in individuals with periodontal inflammation. J. Clin. Periodontol..

[29] Crasta K (2009). Bacteraemia due to dental flossing. J. Clin. Periodontol..

[30] (American Physiological Society, 2014).

[31] Zarate MA, Chang EI, Wood CE (2018). Effects of ketamine on the fetal transcriptomic response to umbilical cord occlusion: Comparison with hypoxic hypoxia in the cerebral cortex. J. Physiol..

[32] Bradford MM (1976). A rapid and sensitive method for the quantitation of microgram quantities of protein utilizing the principle of protein-dye binding. Anal. Biochem..

[33] Conesa A (2016). A survey of best practices for RNA-seq data analysis. Genome Biol..

[34] Grabherr MG (2011). Full-length transcriptome assembly from RNA-Seq data without a reference genome. Nat. Biotechnol..

[35] Bryant DM (2017). A tissue-mapped axolotl de novo transcriptome enables identification of limb regeneration factors. Cell Rep..

[36] Langmead B, Salzberg SL (2012). Fast gapped-read alignment with Bowtie 2. Nat. Methods.

[37] Bolger AM, Lohse M, Usadel B (2014). Trimmomatic: A flexible trimmer for Illumina sequence data. Bioinformatics.

[38] Li B, Dewey CN (2011). RSEM: Accurate transcript quantification from RNA-Seq data with or without a reference genome. BMC Bioinform..

[39] Robinson MD, McCarthy DJ, Smyth GK (2010). edgeR: A Bioconductor package for differential expression analysis of digital gene expression data. Bioinformatics.

[40] Gregory R. Warnes [aut], B. B. a., Lodewijk Bonebakker [aut], Robert Gentleman [aut], Wolfgang Huber [aut], Andy Liaw [aut], Thomas Lumley [aut], Martin Maechler [aut], Arni Magnusson [aut], Steffen Moeller [aut], Marc Schwartz [aut], Bill Venables [aut], Tal Galili [ctb, cre].

[41] Stelzer, G. *et al.* The GeneCards suite: From gene data mining to disease genome sequence analyses. *Curr. Protoc. Bioinform.* **54**, 1.30.31–31.30.33. 10.1002/cpbi.5 (2016). 10.1002/cpbi.5 27322403

[42] Maere S, Heymans K, Kuiper M (2005). BiNGO: A Cytoscape plugin to assess overrepresentation of gene ontology categories in biological networks. Bioinformatics.

[43] Shannon P (2003). Cytoscape: A software environment for integrated models of biomolecular interaction networks. Genome Res..

[44] Wang J, Duncan D, Shi Z, Zhang B (2013). WEB-based GEne SeT AnaLysis Toolkit (WebGestalt): update 2013. Nucleic Acids Res..

[45] Warde-Farley D (2010). The GeneMANIA prediction server: Biological network integration for gene prioritization and predicting gene function. Nucleic Acids Res..

[46] Ye J (2012). Primer-BLAST: A tool to design target-specific primers for polymerase chain reaction. BMC Bioinform..

[47] Kim TK (2015). T test as a parametric statistic. Korean J. Anesthesiol..

[48] McKnight PE, Najab J (2010). Mann–Whitney U Test. Corsini Encycl. Psychol..

[49] B., C. A. Normal rectal temperatures of sheep. **85**, 251–270 (1928).

[50] Walker MG, Volkmuth W, Sprinzak E, Hodgson D, Klingler T (1999). Prediction of gene function by genome-scale expression analysis: Prostate cancer-associated genes. Genome Res..

[51] Wu LF (2002). Large-scale prediction of *Saccharomyces cerevisiae* gene function using overlapping transcriptional clusters. Nat. Genet..

[52] Zhang W (2004). The functional landscape of mouse gene expression. J. Biol..

[53] Aagaard K (2014). The placenta harbors a unique microbiome. Sci Transl Med.

[54] Stout MJ (2013). Identification of intracellular bacteria in the basal plate of the human placenta in term and preterm gestations. Am. J. Obstet. Gynecol..

[55] Borghi E (2018). Antenatal microbial colonization of mammalian gut. Reprod. Sci..

[56] Han YW (2006). Transmission of an uncultivated Bergeyella strain from the oral cavity to amniotic fluid in a case of preterm birth. J. Clin. Microbiol..

[57] Han YW, Shen T, Chung P, Buhimschi IA, Buhimschi CS (2009). Uncultivated bacteria as etiologic agents of intra-amniotic inflammation leading to preterm birth. J. Clin. Microbiol..

[58] Han YW (2010). Term stillbirth caused by oral *Fusobacterium nucleatum*. Obstet. Gynecol..

[59] Su M (2018). Diversified gut microbiota in newborns of mothers with gestational diabetes mellitus. PLoS ONE.

[60] Gomez de Agüero M (2016). The maternal microbiota drives early postnatal innate immune development. Science.

[61] agiiero, m. g. d.

[62] Prince AL (2016). The placental membrane microbiome is altered among subjects with spontaneous preterm birth with and without chorioamnionitis. Am. J. Obstet. Gynecol..

[63] Swati P (2012). Simultaneous detection of periodontal pathogens in subgingival plaque and placenta of women with hypertension in pregnancy. Arch. Gynecol. Obstet..

[64] de Goffau MC (2019). Human placenta has no microbiome but can contain potential pathogens. Nature.

[65] Kershaw CM (2005). The anatomy of the sheep cervix and its influence on the transcervical passage of an inseminating pipette into the uterine lumen. Theriogenology.

[66] Guyton, A. C. & Hall, J. E. *Medical Physiology*. 11 edn., 1011 (Elsevier Saunders, Amsterdam).

[67] Ferenczy, A. In *Pathology of the Female Genital Tract* (ed. Ancel, B.) 102–123 (Springer, New York, 1977).

[68] Naqvi SMK (2005). Evaluation of gross anatomical features of cervix of tropical sheep using cervical silicone moulds. Anim. Reprod. Sci..

[69] Benziger DP, Edelson J (1983). Absorption from the vagina. Drug Metab. Rev..

[70] Wood TK, Knabel SJ, Kwan BW (2013). Bacterial persister cell formation and dormancy. Appl. Environ. Microbiol..

[71] Hao R (2013). Proteasomes activate aggresome disassembly and clearance by producing unanchored ubiquitin chains. Mol. Cell.

[72] Balana ME (2005). ARF6 GTPase controls bacterial invasion by actin remodelling. J. Cell Sci..

[73] Gillman AN (2017). Epidermal growth factor receptor signaling enhances the proinflammatory effects of *Staphylococcus aureus* gamma-toxin on the mucosa. Toxins (Basel)..

[74] Slanina H, Mundlein S, Hebling S, Schubert-Unkmeir A (2014). Role of epidermal growth factor receptor signaling in the interaction of *Neisseria meningitidis* with endothelial cells. Infect. Immun..

[75] Romero R (1988). Labor and infection. II. Bacterial endotoxin in amniotic fluid and its relationship to the onset of preterm labor. Am. J. Obstet. Gynecol..

